# LOWER-EXTREMITY CONSTRAINT-INDUCED MOVEMENT THERAPY IN INDIVIDUALS WITH STROKE – IMPROVEMENTS, EXPERIENCES AND HEALTH-RELATED QUALITY OF LIFE

**DOI:** 10.2340/jrm-cc.v8.42829

**Published:** 2025-04-01

**Authors:** Ingela Marklund

**Affiliations:** From the Department of Community Medicine and Rehabilitation, Umeå University and Centre for Clinical Research and Education, Region Värmland Karlstad, Sweden


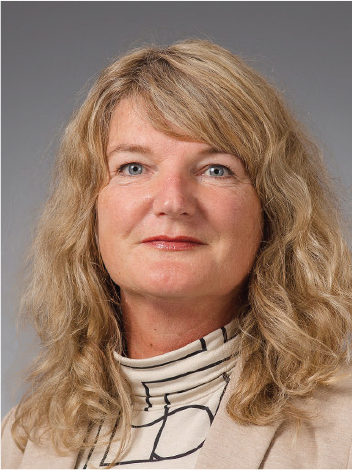
 On November 24, 2023 Ingela Marklund defended her thesis Lower-extremity constraint-induced movement therapy in individuals with stroke – improvements, experiences and health-related quality of life at the Umeå University, Umeå, Sweden. Supervisors: Maria Klässbo, Brynjar Fure, Britt-Marie Stålnacke, Xiaolei Hu.

Main findings

- LE-CIMT improves body functions and activities after a stroke, regardless of age, sex, stroke type or affected side.
- Achieved improvements persisted over time.
- Intensive treatment inspires hope by demonstrating possibilities for change, enhancing independence and self-esteem.
- HRQoL remains reduced in physical functioning, role-functioning (physical), general health and social functioning compared to the general population.
- Walking distance is associated with physical functioning in HRQoL, highlighting the importance of mobility training in poststroke rehabilitation.

Stroke is the third-leading cause of disability worldwide, and there are rehabilitation needs not only in the first year but also throughout the lifetime. The ability to walk is crucial in everyday life since it affects mobility, self-care and social activities. National guidelines recommend treating impairments with repetitive task- and goal-oriented exercises. One form of highly intensive and task-specific treatment is constraint-induced movement therapy (CIMT), a treatment method developed based on understanding brain plasticity with a behavioural explanatory model. There is insufficient evidence regarding CIMT for the lower extremities (LE-CIMT) since it has only been investigated in a few published studies involving only a small number of participants. The overall aims of this thesis were to explore the extent to which LE-CIMT (6 h per day for 2 weeks) can improve impaired body functions and limited activities, describe how the treatment is experienced by patients and investigate whether it affects the health-related quality of life (HRQoL) of individuals with stroke. This thesis contains 5 papers that analysed data from 2 study populations using quantitative and qualitative research methods (Table I).

**Table I:** Overview of the 5 studies included in this thesis

Paper	I	II and III	IV	V
Method	Single-subject experimental design, AB phase, and follow-up	Longitudinal uncontrolled cohort study with follow-up	Qualitative semi-structured interviews	Cross-sectional survey
Main research questions	Can LE-CIMT improve motor function, balance, functional mobility, strength and walking ability in clinical practice		Describe participants experiences of LE-CIMT	Describe participants health-related quality of life compared to norm-based data
Participants, *n*	5	147	7	106
LE-CIMT	Torsby Hospital	Katarina- and Liljeholms-kliniken	Katarina-kliniken	Katarina- and Liljeholms-kliniken
Age, years	62 (46–81)	51 (20–72)††	55 (35–74)	62 (26–89)
Sex, male/female	2/3	100/47	4/3	69/37
More-affected leg, right/left	2/3	84/62^†^	3/4	62/44

Data are presented as mean (range) or *n*. ^†^One missing value. ^††^Three missing values.

CIMT: constraint-induced movement therapy.

The intervention consisted of individually tailored LE-CIMT in groups of 2–4 participants with intensive massed practice (6 h/day for 10 consecutive weekdays) supervised by a physiotherapist. The LE-CIMT included various training sessions focusing on motor function, balance, strength, locomotor training indoors and outdoors, functional training and stretching of short muscles. Training programmes were based on pre-test results and individual goals and were revised on a daily basis by the physiotherapist. More details about the intervention and data collection are described in the chapters *Intervention* and *Data collection* (Table II).

**Table II:** The measures used in this thesis classified according to the International Classification of Functioning, Disability and Health (ICF).

Outcome measure	ICF	Paper
Fugl–Meyer Assessment Scale	Body function	I–II
Timed Up-and-Go	Activity	I–III
Berg Balance Scale	Body function and activity	III
Single-Leg Stance	Body function	III
Step Test	Body function	I
Ten-Metre Walk Test	Activity	I–II
Six-Minute Walk Test	Activity	I–II
One Repetition Maximum	Body function	III
Weight Distribution	Body function	I
Nine-Hole Peg Test	Activity	I
Qualitative Interviews	Body function, activity, participation, personal and environmental factors	IV
RAND-36	Body function, activity participation and personal factors	V
Saltin–Grimby Physical Activity Level Scale	Activity	V
Questionnaire	Activity, participation and personal factors	V

ICF: The International Classification of Functioning, Disability and Health.

The results showed in paper I that LE-CIMT can improve motor function, dynamic balance, mobility and walking ability in individuals in the chronic stage of stroke recovery. Of the 30 variables measured in 5 subjects with 6 measuring instruments, 23 (77%) improved, with 12 (52%) improving significantly. The improvements remained at the long-term follow-ups (3 and 6 months after LE-CIMT) for 22 of the 23 variables. In addition, the weight-bearing distribution measured standing on 2 scales before, directly after and 3 and 6 months after LE-CIMT. All subjects increased the weight borne by their more affected leg after LE-CIMT, providing a more symmetric weight distribution, and the improvements persisted at the 3- and 6-month follow-ups.

**Fig. 1:**
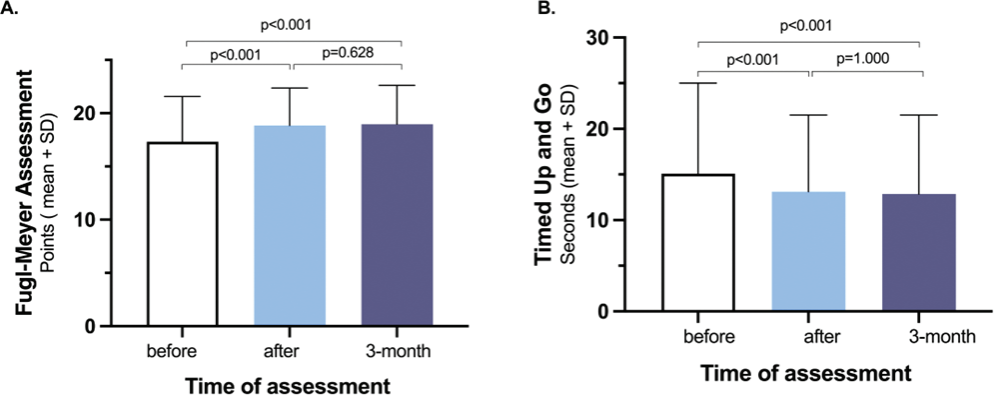
Statistically significant improvements direct- and 3-months after LE-CIMT in the Fugl-Meyer Assessment scale (A) (sub-item voluntary movements, max 22 points) and Timed Up and Go (B) compared to the pre-intervention data.

**Fig. 2:**
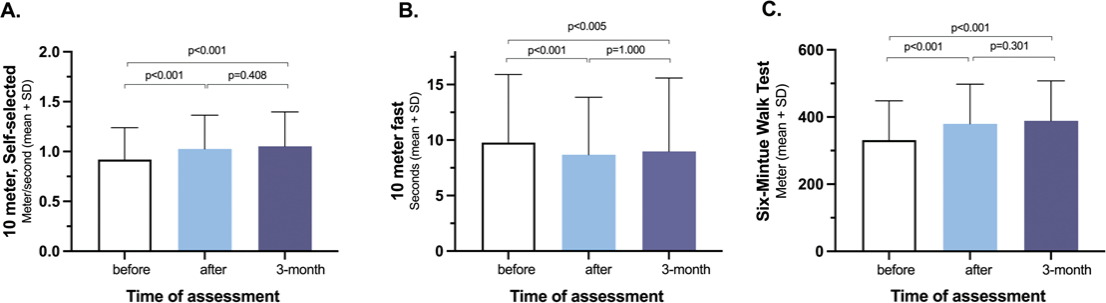
Compared to the pre-intervention data, statistically significant improvements in the 10-metre walk test, self-selected speed (A) 10 meter walk test, as fast as possible (B) 6-minute walk test (C) direct after and 3-months after LE-CIMT.

**Fig. 3:**
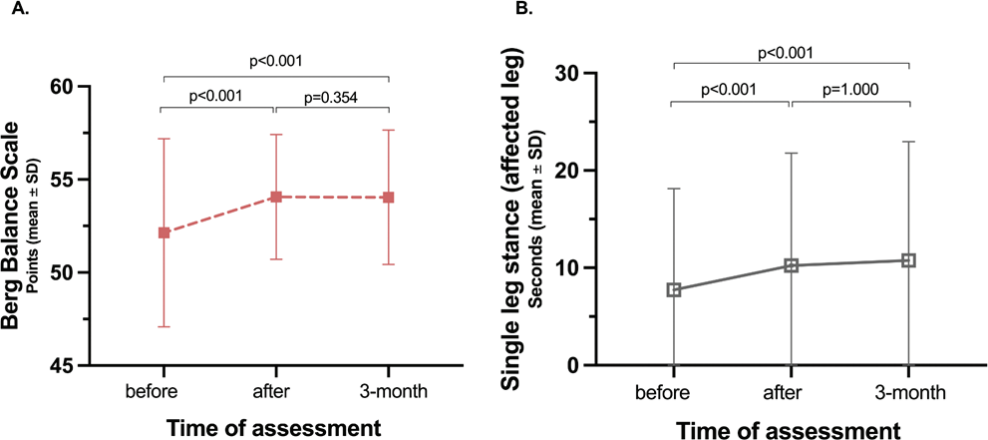
Improvements in balance assessed by the Berg Balance Scale (A) and single leg stance (B).

**Fig. 4:**
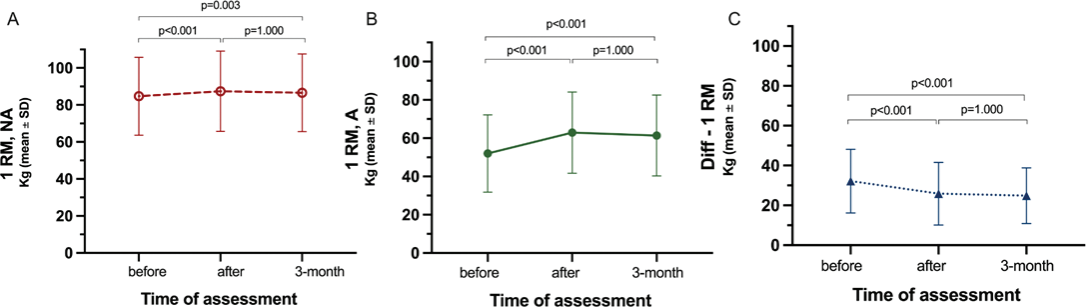
Improvements in leg strength for the (A) non-affected and (B) affected leg and (C) the difference in leg strength between the non-affected and affected legs.

**Fig. 5:**
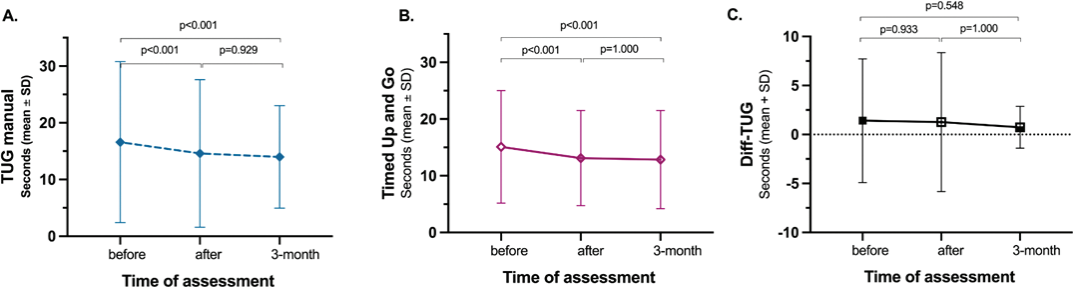
Results for dual task (C) assessed by the (A) TUG Manual test compared with the (B) TUG test.

In papers II and III, LE-CIMT significantly enhanced motor function, mobility, balance, dual-task ability, leg-strength and walking ability. These improvements remained at the 3-month follow-up (Figs 1–5). Those who completed the intervention 1–6 months after stroke onset showed significantly greater improvements in walking speed, measured with 10-m walk test during the follow-up than those who completed the intervention 7 months or more after stroke onset. In addition, balance measured using the Berg balance score scores decreased with age.

In the analysis of the interviews (Paper IV), 2 main categories emerged from the manifest content (*the therapy* and *me and my body*) with 4 and 3 subcategories, respectively. From these categories, an overall theme constituted the latent content of the material: *knowledge of myself and my prospects for leading an easier life.*

The main category (*the therapy*) included the informant’s experiences of the preparation before LE-CIMT, the actual intensive training and its improvements, and their views of the staff who conducted LE-CIMT. Preparation was considered vital for all participants undergoing LE-CIMT. Since LE-CIMT involves rigorous and intense training, it was crucial to mentally prepare for the challenges ahead.

“… Then you had to go to an information meeting and take a relative with you because these relatives must also accept that it’s going to be so tough.” [I:5]

The training was experienced as concentrated, intensive and challenging since each exercise was always at the limit of each individual’s ability and capacity. The restriction, with the whole-leg orthosis, of the less-affected leg during training forced the informants to use the more affected leg more than before. The training consisted mainly of learning, for example, how to walk in a new way without compensation and to do things they did not believe they would manage.

“And then the feeling that you pressurise all your senses and all your muscles so much, and yes, these are really things you’ve never done before.” [I:6]

The physical improvements were experienced during and after LE-CIMT and described in various ways: stronger leg, better balance, better coping and greater use of the leg than before.

“It’s starting to come bit by bit, afterwards, now; and above all, I feel my right leg is as strong as my left, so I can rely on it more.” [I:3]

A feeling of freedom outweighed all the hard work, and the informants became aware of how fragile and exposed their situation was.

The informants reported that the physiotherapists were very professional, competent and sensitive to changes. They made demands, provided positive feedback and encouragement, participated in all activities during the day and “shared their lives.”

“They join in, and we eat together, and they join in all the time, and they’re on to you; you can hardly cheat on anything without them turning up.” [I:7]

The second main category (*me and my body*) included the informants’ reflections on their importance in the treatment, how this has affected them and what insight it provided about their situation. The informants’ motivation, persistence and the fact that they decided to participate were critical aspects of completing LE-CIMT. They felt that they were specially chosen and made an effort since few had the opportunity to undergo this therapy.

“I think you have to be selfish enough to focus on yourself.” [I:4]

The physical improvements changed the informants’ perspective of their capacity and provided a sense of human dignity, “not to be finished as a human being.” By succeeding in various exercises and achieving their goals, self-esteem was strengthened, and faith in the future was awakened. The knowledge that informants gained about their bodies during treatment gave them security in their everyday lives. Change is also about spreading knowledge and the experience they have gained. It was important for them to be able to talk about their experiences and thus influence the situation of others recovering after a stroke. They also perceived that others experienced them differently after LE-CIMT.

“There’s a lot of, I think, positive things about working hard, too, partly because you get attention yourself and the help you need, but in this way, you also get past these natural obstacles, you believe you’re going to manage things you need, but in this way, you also get past these natural obstacles, you believe you’re going to manage things yourself. You grow in yourself, you recover a certain position in your home.” [I:4]

The informants felt frustrated that few in healthcare knew about LE-CIMT. Reflecting on their earlier rehabilitation, the informants expressed a need to “raise the bar” and for an overarching programme that begins at acute stroke onset and encompasses lifelong rehabilitation so they can plan their future.

“I can’t understand how they think. First, they must rescue a person … I mean, we might well have died … but then they sit there in the care system and save them, and then they tell you you must live, but you don’t get any help for living.” [I:7]

Despite their knowledge and experience of LE-CIMT, the informants felt they could not train intensively by themselves, and recurring periods of LE-CIMT were needed. The frustration that LE-CIMT is unavailable in healthcare and the experience that the county councils resist introducing new treatment methods became clear.

“But can’t you pay, I asked, to get this therapy (LE-CIMT), but that was not possible either.” [I:1]

The informants experienced that LE-CIMT, through intensive repetition of training and information/education, gave them knowledge about themselves and how their body works, allowing them to live an easier life. They felt there was still hope and opportunity for functional improvements, which increased their independence and self-esteem.

“But when I look back on CIMT, I see that … it was this that, in fact, change everything; it made things really great.” [I:5]

In paper V, the respondents estimated lower HRQoL than norm-based data in every domain except pain where it was equal. The greatest differences were seen in the physical functioning and role-functioning physical domains. Comparisons between the sample’s means and norm-based reference data showed significantly decreased HRQoL for the participants in 4 out of 8 domains in the Swedish RAND-36 scale, physical functioning, role-functioning physical, social functioning and general health. There were no significant differences in HRQoL for the domains pain, energy/fatigue and role-functioning emotional well-being. In the univariable analysis adjusted for age and sex, the linear relationship between participants’ scores in the 8 RAND-36 domains and their previous result on walking distance measured with Six-minute Walk test (6MWT), time since treatment and living conditions were analysed. There was a significant association between 6MWT and physical functioning. For every 100-m increase in 6MWT after LE-CIMT, the physical functioning score increased by 6.45 points in RAND-36. No other significant associations were found. More details about the results in the various papers are described in the chapter *Results*.

Finally, Chapter *Discussion* provides a general discussion of the results of this thesis. The main conclusion is that LE-CIMT appears useful in improving impaired body functions and limited activities after a stroke and can be conducted in day hospital rehabilitation and outpatient clinics. It seems possible to achieve these improvements even a long time after stroke onset, regardless of age, sex, stroke type or affected side. Earlier treatment after stroke onset generates greater improvements in walking ability, and the improved dual-task ability might reduce the risk of falls. LE-CIMT also provides knowledge and how to live an easier life. The intensive treatment gave hope since the improvements showed the possibilities for change, which, in turn, increasing independence and self-esteem. Whilst LE-CIMT was hard and difficult, it was felt entirely necessary.

“This is what my body needed.” [I:6]

The general discussion examines the results in the thesis in relation to previous studies, methodological considerations, strengths and limitations during study design, data collection and statistical analysis. Finally, the thesis considers the clinical implications and possible directions for future research.
